# Living well with dementia: What is possible and how to promote it

**DOI:** 10.1002/gps.5627

**Published:** 2021-10-13

**Authors:** Catherine Quinn, James A. Pickett, Rachael Litherland, Robin G. Morris, Anthony Martyr, Linda Clare, On behalf of the IDEAL Programme Team

**Affiliations:** ^1^ Centre for Applied Dementia Studies, Faculty of Health Studies University of Bradford Bradford UK; ^2^ Wolfson Centre for Applied Health Research Bradford UK; ^3^ Health Data Research UK London UK; ^4^ Innovations in Dementia Exeter UK; ^5^ King's College Institute of Psychiatry, Psychology and Neuroscience London UK; ^6^ REACH: The Centre for Research in Ageing and Cognitive Health, College of Medicine and Health University of Exeter Exeter UK; ^7^ NIHR Applied Research Collaboration South‐West Peninsula Exeter UK

**Keywords:** Alzheimer's, carer, post‐diagnostic support, quality of life, well‐being

## Abstract

- The focus on living well with dementia encourages a more positive and empowering approach
- The right support can improve the experience of living with dementia
- An holistic approach to assessing the needs of people with dementia and identifying the factors that impact on their well‐being is essential
- Enabling people to live better requires a broad approach that encompasses both health and social systems and the wider community

## 1 INTRODUCTION

Dementia is a syndrome that predominantly impacts on cognitive functions and everyday activities.^1^, ^2^ There are different sub‐types of dementia which can have different causes. The progression of dementia will vary between individuals and people will experience it in different ways, but it typically affects memory, thinking, language, orientation and judgement and social behaviour.^3^, ^4^ Dementia is a progressive condition and the symptoms will change over the years. Estimates indicate that worldwide there were 50 million people living with dementia in 2019.^5^ Currently there are no treatments that will either cure nor prevent the progressive course of the disease,^4^ although for some people medication can slow the progression of dementia. However, it is possible to improve the lives of those affected by dementia.^4^

The last decade has seen a radical worldwide effort to shift the framing of dementia policy from a fatalistic perspective concentrating on the negative consequences of dementia as a disorder to a more positive and empowering narrative that seeks to prioritise quality of life (QoL). This has broadened the focus to encompass not only medical approaches such as pharmacological treatments, but also the recognition that dementia is a manageable condition, given proper support. However, the move towards creating positive narratives for people with dementia has been contentious. Viewing dementia as a condition that people manage and live with may reduce stigma and promote a more positive sense of identity. However, the terminology of ‘living well’ with dementia, whilst aspirational, can be regarded as unachievable for many,^6^ failing to recognise the realities of the high levels of support needed to make dementia a manageable condition. Here we explore the subjective and objective experiences of people with dementia to develop a better understanding of what living well might mean in practice. Drawing on empirical research studies and input from people with dementia, we propose recommendations for clinical practice that may support people to live as well as possible with a diagnosis of dementia.

## 2 ‘LIVING WELL’ WITH DEMENTIA – THE POLICY CONTEXT

Recently, the focus on treating the medical symptoms of dementia has shifted to a more holistic and person‐centred approach that supports and enables people to live with the condition, reflected in international policies and guidelines. It has been recognised that to provide appropriate support for the complex needs of people with dementia requires a multi‐stakeholder approach involving the health and social care system and other governmental sectors. Supporting these developments is a recognition that dementia is a public health priority.^4^ This has led to calls for 75% of countries to have developed or updated national policies, strategies, plans or frameworks for dementia by 2025.^7^ These should be developed in conjunction with people with dementia and other relevant stakeholders.^8^ A number of countries have developed national dementia plans to address the needs of those affected by dementia, which are tailored to the culture of each country. A review of 21 national dementia strategies and two national neurodegenerative strategies from countries in Europe identified differences in approaches. However, there were commonalities in key areas such as raising awareness and improving care and treatment for people with dementia.^9^

The title of the current English national dementia strategy, ‘Living Well with Dementia’,^10^ emerged through consultation with people with dementia and carers who acknowledged that whilst a dementia diagnosis is devastating, with appropriate support people can live fulfilling lives.^11^ Thus, the aim was to address a perception that there was nothing that could be done for people with dementia and, following worldwide trends, to change the narrative to one of positive intervention and enablement. The strategy was designed to meet the needs of all people with dementia, regardless of their diagnosis, age, or ethnicity.^12^ It focused on three core areas pertaining to improved awareness, earlier diagnosis and intervention, and a higher quality of care.^10^ The concept of living well is embedded in the Live Well pathway for dementia developed by the national Health Service in England, which focuses on a five‐step journey: preventing well; diagnosing well; supporting well; living well; and dying well.^13^ It is also included as the central facet of national strategies in Canada^14^ and across Europe.^9^ These core messages about living well have been built on by advocacy organisations and charities globally, leading to relevant developments at community level.^15^

## 3 THE ‘VOICE’ OF THOSE LIVING WITH DEMENTIA

This shift of focus towards enabling people to live well or have a good QoL has in part been driven by people with dementia themselves. Most powerfully, people living with a diagnosis of dementia are speaking out about their experiences, creating alternative narratives about what it means to live with dementia and increasing public understanding of the condition.^16^, ^17^ With a policy shift to a focus on earlier diagnosis of dementia it is increasingly possible to hear the previously unheard voice of people with early signs of dementia.^18^ By sharing their stories this can bring enlightenment to those who hear them. These stories can also counter the often negative portrayal of dementia,^19^ for example discourses in the media focussing on the ‘catastrophic’ nature of dementia or that it was ‘worse than death’.^20^ Whilst these narratives provide a positive contribution to our understanding of dementia, it is important to consider which voices are absent from these narratives and hence currently unheard, in order to ensure a comprehensive understanding that reflects the experience of all those living with dementia.^19^

There has also been the emergence of a human rights‐based perspective, reminding us that people with dementia are ‘citizens’ with a right for their voices to be heard.^21^, ^22^ In the UK, this rights‐based approach has been ingrained in the Dementia Statements put forward by the National Dementia Action Alliance; developed by people with dementia and carers, these highlight ways in which people should not be treated differently because of their diagnosis.^23^ These statements, listed in Box 1, highlight elements that people with dementia and carers identify as being important to their lives.

**BOX 1 The dementia statements**

- Identity. We have the right to be recognised as who we are, to make choices about our lives including taking risks, and to contribute to society. Our diagnosis should not define us, nor should we be ashamed of it.
- Community. We have the right to continue with day‐to‐day and family life, without discrimination or unfair cost, to be accepted and included in our communities and not live in isolation or loneliness.
- Carers. We have the right to be respected, and recognised as partners in care, provided with education, support, services, and training which enables us to plan and make decisions about the future.
- Care. We have the right to an early and accurate diagnosis, and to receive evidence‐based, appropriate, compassionate and properly funded care and treatment, from trained people who understand us and how dementia affects us. This must meet our needs, wherever we live.
- Research. We have the right to know about and decide if we want to be involved in research that looks at cause, cure and care for dementia and be supported to take part.

## 4 LIVING WELL WITH DEMENTIA – UNPICKING THE ASPIRATION

The emphasis on enabling people to live well with dementia raises two key questions: what does it actually mean to live well and can we effectively measure a person's ability to live well?

The capacity to live well with chronic illness and disability has been defined as experiencing ‘the best achievable state of health that encompasses all dimensions of physical, mental, and social well‐being’ and it is acknowledged that ‘living well is shaped by the physical, social, and cultural surroundings and by the effects of chronic illness’ (p. 32).^24^ This suggests that living well is a multi‐faceted construct, influenced by the person's physical and psychological wellbeing and wider social environment. We can think of living well as multi‐faceted, similar to concepts of successful ageing.^25^ Thus we should not be categorising people as ‘living well’ or not ‘living well’ but instead identifying where they are on this spectrum.

Typically living well has been equated to experiencing a good QoL, which is the most commonly used method of assessing living well.^26^ Although perceived QoL is an important indication of whether a person is living well, it may not fully encapsulate all elements of the continuum. The approach taken by Clare and colleagues has been to perceive living well as a combination of constructs: QoL, well‐being and life satisfaction.^27^ This is supported by the results of multivariate analyses.^28^ Utilising this approach taps into the multi‐dimensional structure of living well and is a positive move away from using single measures. In this emerging area, further work is still needed to understand how to most effectively measure living well.

## 5 WHAT DOES LIVING WELL MEAN TO PEOPLE WITH DEMENTIA?

A focus on enabling people to live well needs to consider the way in which people with dementia and carers themselves define and understand living well. Living well will mean different things to different people and expectations will relate to individual circumstances. For example, what is required to enable someone to live well in the early stages of dementia may be very different in the later stages. People with dementia, particularly in the early stages, have articulated what it means to live well and tend to have very individualised definitions. These to some extent relate to how they lived their lives before developing dementia and a desire to continue this lifestyle. The following comments recorded from people with dementia taking part in Time 1 of the IDEAL programme illustrate this individualisation:

Living well is relative to my current situation, sometimes it's about compensating for things such as lack of energy. Living well is about meeting competing demands of what I want to do, what I can do, and taking part in family life.

Being able to do all my usual things and go out walking, I use lists to help with this. Keeping in contact with friends and family, don't lock yourself away. I still try to do everything I used to do even if it's not perfect.

The ability to get out and about and carry on an active place in society. Overcoming various health problems the best that I can and finding enjoyment in life, as opposed to sitting and vegetating. I live by the phrase ‘I may have dementia, but dementia doesn't have me’.

Equally, it is clear that some individuals do not consider themselves to be living well:

I don't think I'm living well anymore. I can't do what I want to do … less capable at doing things, organising skills have been lost.

Living well would mean getting independence back and get out and start living again. Feels like it's gone from everything to nothing.

I can't get out on my own, if I could, [I] could live better. I have to depend on people.

These quotes illustrate some of the different areas that are considered important for living well. Equally, they demonstrate that a person's capability to live well is not purely defined by the impact the diagnosis of dementia is having on that person's life. There were some who did not feel as if they were living well, identifying the loss of abilities and independence. This highlights the need for people to receive appropriate support to enable them to live well as in some of these cases they could have been helped to retain some level of independence.

The issues covered in the quotes are also evident in the findings presented in Table 1 which illustrates key areas identified by 1339 people with mild‐to‐moderate dementia taking part in Timepoint 1 of the IDEAL programme^27^, ^29^ responding to the question ‘What does living well mean to you?’

**TABLE 1:** Summary of responses to the question ‘What does living well mean to you’ by 1339 people with dementia in the IDEAL cohort

Category	Number of references coded
Engaged and active lifestyle	979
Positive relationships with others	636
Good living situation and environment	408
Having security	388
Getting on with life	318
Being able to get out and about	250
A positive outlook on life	236
Being able to cope	194
Having independence	155
Having a purpose	112
Unsure	25

*Note*: Numbers equate to number of references coded; some responses are coded under more than one category.

These responses primarily draw on psychosocial aspects such as relationships with others, living situation, and outlook on life. Having an engaged and active lifestyle involved people being healthy and being able to partake in exercise or hobbies. Positive relationship involved not only having good quality relationships with others but also social contact. Living well was also defined by people's living situation and whether they were living in good housing, in communities where there were local amenities and things to do. Related to this people also described a need to be able to ‘get out and about’ which involved being able to get out the house or go on trips or holidays. They also needed appropriate transportation to enable them to be able to get out, so access to cars or public transportation was important. Living well was also akin to just being able to get on with life and having an enjoyable life with no concerns.

Whilst the term living well was seen as a shift towards a more positive framing of dementia, different interpretations of this term have created some challenges. Some people with dementia may feel it is not possible to live well. Others may feel that living well is something that they have to aspire to or achieve:

On… foggy days… I do find it harder to live up to the expression ‘living well with dementia’, and find this on those days to be a burden rather than an incentive.^16^

This raises the question of responsibility for ‘living well’. Should the onus be on people with dementia and/or carers to ensure that they are living well? Whilst national policies focus on the development of health and social care services, there is clearly a need for society as a whole to support people affected by dementia. A better understanding of the factors that can influence whether someone is ‘living well’ can lead to more effective support for people with dementia.

## 6 WHAT MIGHT INFLUENCE A PERSON'S CAPABILITY TO LIVE WELL WITH DEMENTIA?

In addition to exploring what aspects of living well are important to people with dementia, studies have investigated factors that are related to, or might influence, the potential for living well. The evidence‐base is somewhat dominated by the use of QoL as an outcome measure; a plethora of factors have been linked to QoL in dementia, but many of these associations have been small or negligible.^26^ Here we draw on evidence from a meta‐analysis of observational studies of QoL in dementia,^26^ a meta‐synthesis of qualitative studies on QoL from the perspective of people with dementia,^30^ and cross‐sectional findings from a large‐scale cohort study of factors linked to living well in people with mild‐to‐moderate dementia.^28^ From this evidence there appear to be six key domains robustly linked to living well or QoL.

The first is the person's psychological characteristics and health, which includes their outlook on life.^26^, ^28^, ^30^ In the cohort study,^28^ this factor in particular was most strongly linked to the capability to live well, indicating that as well as managing physical symptoms people need support to maintain their psychological health. The second factor is the person's level of physical fitness and physical health,^26^, ^28^ although this was not identified in the qualitative accounts of people with dementia.^30^ The third is the person's level of social engagement and connectedness with others and the environment, including social networks.^26^, ^28^, ^30^ The fourth is the person's ability to have independence in daily activities, to manage everyday life, and carry out activities of daily living.^26^, ^28^, ^30^ The fifth concerns the person's relationships with others, the quality of these relationships, and feelings of loneliness.^26^, ^28^, ^30^ Last is the person's perceived social standing and perceived role in society.^28^, ^30^ This association was not identified in the meta‐analysis^26^ as this concept was not measured in enough studies to be included in the review, suggesting a dearth of research in this area. For those people with dementia who have carers it is important to consider the influence of the carer on the person's capability to live well. For example, characteristics of the carer, such as stress levels, reports of caregiving burden, and feelings of competence can influence the person's capability to live well.^26^, ^31^, ^32^

There is growing evidence that factors important for living well in people with dementia are equally important for living well in carers, although there are some differences. Here we draw on evidence from a systematic review of qualitative and quantitative studies on factors linked to QoL in carers of people with dementia^33^ and cross‐sectional findings from a large‐scale cohort study of living well in carers of people with dementia.^34^ Similar to the findings for people with dementia, the first factor was the carer's psychological characteristics and psychological health.^33^, ^34^ In the cohort study^34^ this has the strongest association with living well and equally in the review paper^33^ this had the most consistent association. The second factor is the carer's level of physical fitness and physical health state.^33^, ^34^ The third factor concerns the carer's experience of caregiving, which encompasses carer stress, burden, feelings of role captivity, and the support received from others.^33^, ^34^ The fourth factor concerns the quality of the relationship between the carer and person with dementia.^33^, ^34^ The last factor relates to the carer's social networks, social resources and participation in leisure and cultural activities.^33^, ^34^

These findings clearly demonstrate that it is not just dementia and the associated symptoms that are affecting living well for both people with dementia and carers. Thus, interventions and strategies focused on enabling people to live well need to move beyond simply managing the symptoms of dementia to take into account these other identified domains.

## 7 RECOMMENDATIONS FOR CLINICAL PRACTICE

The concept of living well has become ingrained in dementia strategies but until now there had been no evaluation of the concept and its implication for the treatment of those affected by dementia. Within healthcare often the focus is on instilling hope and providing treatment that either cures or mitigates the person's symptoms. Yet, as dementia is a progressive degenerative condition, clinicians may feel either that there is little that can be done to help or that any help is futile as the condition will only worsen. Focusing on enabling people to live well acknowledges that it is possible for people to live with dementia and indeed live ‘better’ with dementia. However, for this to happen people require effective support. Here we present key recommendations for healthcare professionals:

- An holistic approach to assessing a person's needs is important. From a healthcare perspective enabling someone to live well needs to go beyond just managing the symptoms of dementia, to look at that person and what impacts on their well‐being.
- Ill‐being is not an inevitable consequence of dementia. Whilst receiving a diagnosis of dementia can have a devastating impact, most people will adjust to their diagnosis. The evidence‐base indicates that many factors can influence a person's capability to live well and indeed many of these factors, such as psychological health, can be treatable with appropriate intervention and support.
- Healthcare professionals have a key role both in raising awareness about dementia and in signposting people towards support services. It is important that healthcare professionals are aware of the services available in their area so that they can direct people with dementia and carers to them.
- There needs to be a collectivist approach to enabling people to live well and a wider policy framework must also be in place to ensure this happens. There needs to be a shift from an individualistic perspective to a broader recognition of the importance of ensuring wider societal, health, and social care systems can enable people with dementia to live well.

## 8 CONCLUSION

The focus on enabling people to live well with dementia has encouraged a more positive and empowering approach. However, it is clear that in order for people to live well with dementia they need appropriate support. This requires a broad approach that encompasses governmental policies, health and social systems, and the wider community. Living well will mean different things to different people and there needs to be a holistic approach to assessing the needs of people with dementia and identifying the factors that impact on their well‐being. This will lead to more effective and individualised interventions and support to enable people to live ‘better’ with dementia.

## CONFLICT OF INTEREST

The authors declare no competing interests.

## AUTHOR CONTRIBUTIONS

The article arose from discussions between co‐authors on the concept of living well with dementia and the implications of this for those affected by dementia. Catherine Quinn and James A. Pickett drafted the original manuscript. Rachael Litherland, Robin G. Morris, Anthony Martyr, and Linda Clare critically reviewed, revised, and approved the final version. Catherine Quinn is the guarantor.

## Data Availability

IDEAL data were deposited with the UK data archive in April 2020 and will be available to access from April 2023. Details of how the data can be accessed after that date can be found here: https://reshare.ukdataservice.ac.uk/854293/.
